# Solvatomorphic Diversity in Coordination Compounds of Copper(II) with l-Homoserine and 1,10-Phenanthroline: Syntheses, Crystal Structures and ESR Study

**DOI:** 10.3390/molecules29235621

**Published:** 2024-11-27

**Authors:** Darko Vušak, Marta Šimunović Letić, Marina Tašner, Dubravka Matković-Čalogović, Jurica Jurec, Dijana Žilić, Biserka Prugovečki

**Affiliations:** 1 Department of Chemistry, Faculty of Science, University of Zagreb, Horvatovac 102A, HR-10000 Zagreb, Croatia; dvusak@chem.pmf.hr (D.V.); marta.simunovic12@gmail.com (M.Š.L.); mtasner@chem.pmf.hr (M.T.); dubravka@chem.pmf.hr (D.M.-Č.); 2 Laboratory for Magnetic Resonances, Division of Physical Chemistry, Ruđer Bošković Institute, Bijenička Cesta 54, HR-10000 Zagreb, Croatia; jurica.jurec@irb.hr (J.J.); dzilic@irb.hr (D.Ž.)

**Keywords:** crystal structures, ESR spectroscopy, magnetism, single-crystal X-ray diffraction, solid state, solvatomorphism, structural transformations

## Abstract

In this study, we report the syntheses, crystal structures and magnetic properties of ternary copper(II) coordination compounds with l-homoserine (l-Hhser) and 1,10-phenanthroline (phen). Six new coordination compounds were obtained: [Cu(l-hser)(H_2_O)(phen)]_2_SO_4_·5H_2_O (**1·5H_2_O**), [Cu(μ-l-hser)(H_2_O)(phen)][Cu(l-hser)(H_2_O)(phen)]_3_(SO_4_)_2_∙12H_2_O (**2·12H_2_O**), {[Cu(μ-l-hser)(H_2_O)(phen)][Cu(μ-l-hser)(phen)]SO_4_·6H_2_O}*_n_* (**3·6H_2_O**), {[Cu(μ-l-hser)(H_2_O)(phen)]_2_SO_4_·3H_2_O}*_n_* (**4·3H_2_O**), [Cu(l-hser)(H_2_O)(phen)][Cu(l-hser)(CH_3_OH)(phen)]SO_4_·4H_2_O (**5·4H_2_O**) and {[Cu(l-hser)(CH_3_OH)(phen)][Cu(μ-l-hser)(phen)]SO_4_·5CH_3_OH}*_n_* (**6·5CH_3_OH**)**.** It was shown that slight differences in water content in the synthetic mixtures highly influence the final product, so in some cases, two or three different products were obtained. The compounds were characterized by single-crystal X-ray diffraction and ESR spectroscopy. Crystal packings are based on intensive networks of hydrogen bonds and π interactions. Most water solvent molecules in these microporous compounds are found in discrete pockets (**1∙5H_2_O**, **2∙12H_2_O**, **3∙6H_2_O**, **4∙3H_2_O**). In **5∙4H_2_O**, water molecules are packed in pockets and 1D channels and in **6∙5CH_3_OH** methanol solvent molecules form 1D channels. ESR spectroscopy measured from room down to liquid nitrogen temperature was used for local magnetic characterization of copper centers. The spin Hamiltonian parameters obtained from the spectral simulation revealed copper coordination geometry that is in agreement with the structural results. Furthermore, ESR spectra revealed no significant exchange coupling between copper ions. **3·6H_2_O** showed pronounced antiproliferative activity toward human colon cancer cell lines (HCT116), human breast cancer cell line (MCF-7) and human lung cancer cell lines (H460).

## 1. Introduction

Copper is an essential trace element in humans and is responsible for many biological functions. Its imbalance is strongly associated with Menkes [1], Alzheimer’s [2] and Wilson’s [3] diseases. Copper forms many different coordination compounds, predominantly with oxidation states Cu(II), and rarely Cu(I). In most of the Cu(II) coordination compounds, the coordination number varies from four to six (square-planar, trigonal bipyramidal and octahedral geometries), while there are examples of higher coordination numbers [4,5,6]. In contrast, Cu(I) coordination compounds are mostly four-coordinated with tetrahedral geometry [7]. Copper and copper compounds have shown enormous potential for anticancer applications. Different kinds of copper coordination compounds were constructed and characterized for cancer treatment. Ternary copper coordination compounds (often refered as heteroleptic or three-component coordination compound) with amino acids and heterocyclic bases (1,10-phenanthroline or 2,2′-bipyridine) have been widely investigated due to their excellent in vivo antitumor activity [8,9,10,11,12,13,14]. Among others, Casiopeinas, a group of copper coordination compounds with the formula [Cu(N-N)(α-l-amino acidato)]NO_3_, where N-N is 1,10-phenanthroline or 2,2′-bipyridine, have shown an significant antineoplastic effect and cytotoxicity in cancer cell [15,16]. They can be a starting platform for developing antitumor drugs, but much effort must be addressed to assess the therapeutic effect, biocompatibility and safety of the new copper complexes [17,18]. They are also used as model systems for the research of copper-containing proteins and enzymes [19] and it was discovered that copper coordination compounds with amino acids/peptides and their building units have nuclease activity [13,20,21].

Investigation of solvates of potentially applicable compounds is of great importance since solvates can exhibit diverse chemical properties [22,23,24]. Due to different synthetic approaches and different amino acid side chain coordinating groups, ternary coordination compounds of copper with various amino acids and heterocyclic bases can form different porous structures and different solvatomorphs which are primarily based on hydrogen bonds and π interactions In porous structures the solvent molecules can be in discrete pockets, or can assist in formation of 1D chains, and 2D or 3D networks, while the complex species are most often 0D or 1D coordination species. Porous compounds have attracted great interest because they can recognize and adsorb solvent/gas molecules, which gives them a potential for application in gas or solvent separation or storage [25,26]. In the CSD database, there are 72 data sets of crystallographic data for coordination compounds containing copper(II), aminoacidates and 1,10-phenanthroline, none of which contain l-homoserinate. In most compounds with an aminoacidate and 1,10-phenanthroline, the aminoacidate is coordinated to the Cu atom by the carboxylate oxygen and amino nitrogen atoms forming five-membered chelate rings. Only one of those ternary coordination compounds contains an aminoacidate (aspartate) acting as a tridentate ligand [27]. Although l-homoserine is not a proteinogenic amino acid, it is an important part of metabolism, especially in the biosynthesis of amino acids, such as threonine and methionine [28]. The crystal structure of l-homoserine was first determined by Chacko et al. in 1982 [29], while the crystal-field effect in l-homoserine, as well as the precise hydrogen atoms positions, were reported later [30,31]. There is only one structural report of a coordination compound with l-homoserine, being the ruthenium complex [32]. Our study of the ternary copper coordination compounds with l-homoserine and 1,10-phenanthroline aims to examine synthetic conditions, structural changes, magnetic properties, and anti-cancer activity. Recently we reported the syntheses, crystal structures and solvatomorphic transitions of a series of ternary copper(II) compounds with 2,2′-bipyridine and 1,10-phenanthroline and amino acids glycine, l-alanine, l-valine, l-phenylalanine, l-serine and l-threonine [10,11,12]. In this paper we report the synthesis of six novel solvatomorphs of ternary copper(II) compounds with 1,10-phenanthroline (phen) and l-homoserine (l-Hhser): [Cu(l-hser)(H_2_O)(phen)]_2_SO_4_·5H_2_O (**1·5H_2_O**), [Cu(μ-l-hser)(H_2_O)(phen)][Cu(l-hser)(H_2_O)(phen)]_3_(SO_4_)_2_∙12H_2_O (**2·12H_2_O**), {[Cu(μ-l-hser)(H_2_O)(phen)][Cu(μ-l-hser)(phen)]SO_4_·6H_2_O}*_n_* (**3·6H_2_O**), {[Cu(μ-l-hser)(H_2_O)(phen)]_2_SO_4_·3H_2_O}*_n_* (**4·3H_2_O**), [Cu(l-hser)(H_2_O)(phen)][Cu(l-hser)(CH_3_OH)(phen)]SO_4_·4H_2_O (**5·4H_2_O**) and {[Cu(l-hser)(CH_3_OH)(phen)][Cu(μ-l-hser)(phen)]SO_4_·5CH_3_OH}*_n_*, (**6·5CH_3_OH**). We have performed the reactions of copper(II) sulfate (anhydrous, monohydrate, trihydrate and pentahydrate) with l-homoserine and 1,10-phenanthroline by solution methods (water and/or methanol as a solvent). The effects of solvents on crystallization and crystal structures were investigated for crystal engineering and structure–property relationships. Among other results, our paper gives insight into the structural features of l-homoserine, which may be used for theoretical calculations and investigation of biologically important processes, and its binding modes to copper, an essential metal.

## 2. Results

### 2.1. Synthesis and Crystallization

**Figure 1:**
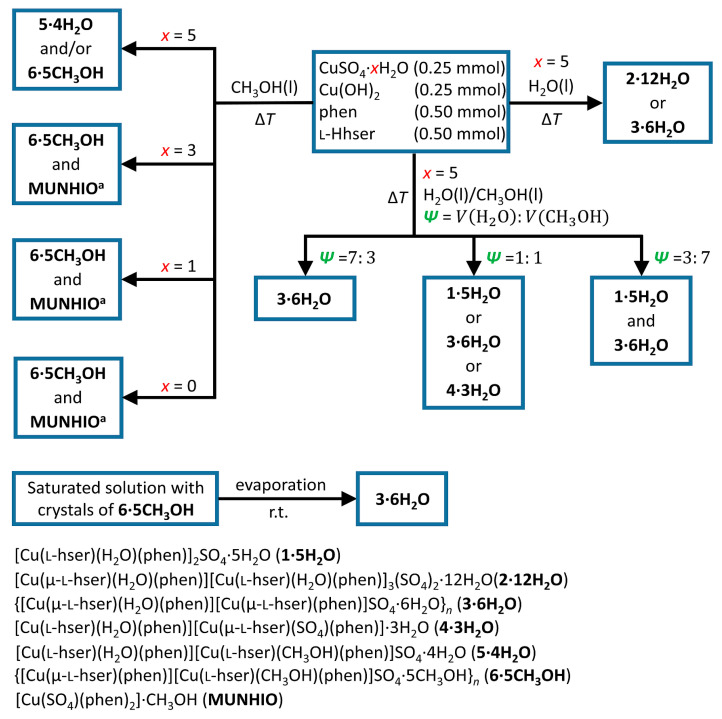
Scheme of the synthetic procedures with the products.

We synthesized six new ternary coordination compounds containing copper(II), 1,10-phenanthroline and l-homoserinate. The results of the synthetic procedures are summarized in Figure 1. Due to the high ratio of solvent molecules within the crystal structures, the solvent has a high influence on the crystallization of the compounds. We obtained different compounds and solvatomorphs by changing the water and methanol ratio in the reaction mixtures. When fully hydrated reactant copper(II) sulfate pentahydrate in pure water was used, blue crystals of **2⋅12H_2_O** or **3⋅6H_2_O** were obtained. After several repeated experiments, we observed that **3∙6H_2_O** formed more often. If the same reactants were used in the mixture of water and methanol (1:1 *v*/*v*), we obtained light blue crystals of **1∙5H_2_O**, blue crystals of **3⋅6H_2_O** or a few blue crystals of **4∙3H_2_O**. In the mixture of water and methanol (7:3 *v*/*v*), only **3∙6H_2_O** is formed, and in the mixture of water and methanol (3:7 *v*/*v*), a mixture of **3∙6H_2_O** and **1∙5H_2_O** is formed. In pure methanol, the same reactants gave **5⋅4H_2_O** and/or **6⋅5CH_3_OH**. In the reactions of less hydrated or anhydrous copper(II) sulfate in methanol, we obtained a mixture of **6⋅5CH_3_OH** and [Cu(SO_4_)(phen)_2_]∙CH_3_OH (CSD refcode MUNHIO) [33].

In most synthetic procedures, we received more than one compound or obtained different products in repeated experiments. The reason behind such diversity is probably the change of conditions during the crystallization process, influencing the stability of different solvates. Lability of the solvates is best observed in Video S1 and Figure S1 in the Supplementary Information. In that experiment, we placed crystals of **6∙5CH_3_OH** immersed into its saturated methanolic solution on a glass holder. As methanol evaporates, the original crystals of **6∙5CH_3_OH** start to dissolve (in the water present in the solution and the surrounding air). After some time, water evaporates, and **3∙6H_2_O** crystallizes on the edges of a drop.

### 2.2. Crystal Structures

**Figure 2:**
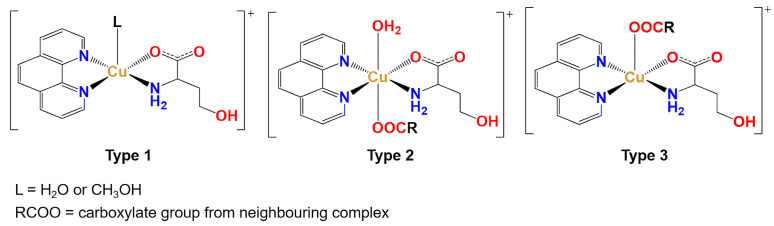
Types of complex species depending on the ligands in the axial positions.

All six newly prepared compounds are ternary coordination compounds containing copper(II), 1,10-phenanthroline and l-homoserinate. Crystallographic data for all six compounds are given in Tables S1 and S2. As explained later in this section, there are some similarities and predictability of the structures’ non-covalent interactions and structural features. Still, there is also some diversity in the coordination sphere. All coordination species contain 1,10-phenanthroline and l-homoserinate, forming two five-membered chelate rings with the copper(II) ion in the equatorial plane. The main difference between the compounds is in the axial positions, so the coordination species can be divided into three types (Figure 2).

Type 1 of the complex species is a square-pyramidal complex cation containing a water or methanol molecule in the apical position with a general formula [Cu(l-hser)(L)(phen)]^+^ (L = H_2_O or CH_3_OH). Type 2, [Cu(µ-l-hser)(H_2_O)(phen)]^+^, is an octahedral complex cation with a water molecule in one axial position and a carboxylate group of the adjacent complex in the other axial position. Type 2 complex cations form either dimers (in **2∙12H_2_O**) or polymeric chains (in **3∙6H_2_O**). Type 3, [Cu(µ-l-hser)(phen)]^+^, is a square-pyramidal complex cation with a carboxylate group from an adjacent complex coordinated in the apical position and is a part of the cationic 1D polymeric chain. The distribution of different types of coordination spheres in crystal structures of compounds is given in Table 1 and Figures S2–S4.

**Figure 3:**
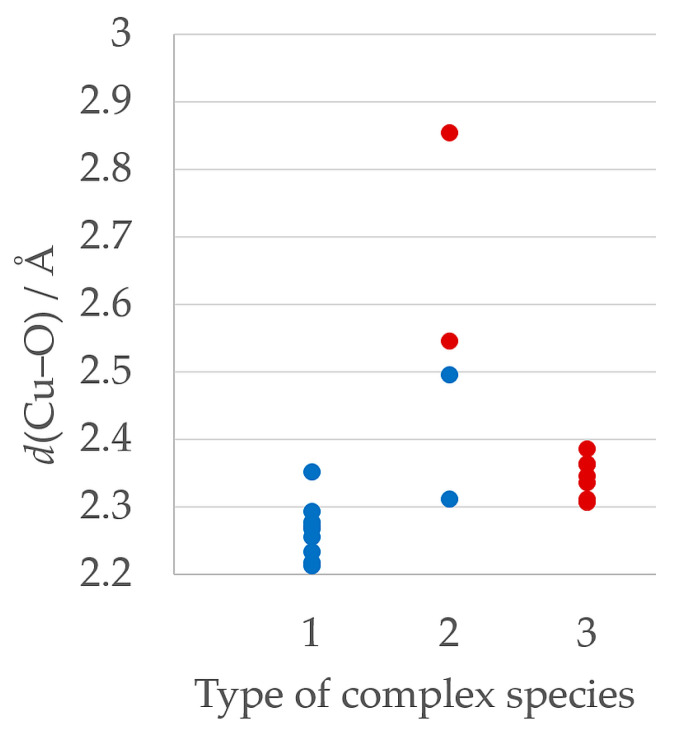
Cu∙∙∙O distances in types 1–3 of complexes in all symmetrically independent complex species. Blue dots represent the distance of copper to the coordinated water or methanol molecule, red dots the distance to the oxygen atom within the carboxylate group.

Cu∙∙∙O and Cu∙∙∙N distances in the equatorial positions are similar in all investigated compounds, but there are some differences in Cu∙∙∙O distances in the axial positions depending on the type of the complex species (Figure 3, Table S3). Cu∙∙∙O distances in type 1, where the oxygen atom originates from the water or methanol molecule, have the shortest distances in the range 2.216(3)–2.353(3) Å. Type 2 has Cu∙∙∙O axial distances in the range 2.313(4)–2.855(4) Å, slightly longer than in types 1 and 3, which can be attributed to *trans-*influence. Most type 3 complex cations exhibit longer Cu∙∙∙O axial distances 2.307(10)–2.387(3) Å compared to type 1. All pentacoordinated complexes (types 1 and 3) exhibit slightly distorted square-pyramidal geometry with *τ*_5_-parameters 0.006–0.198 (Table S4).

**Figure 4:**
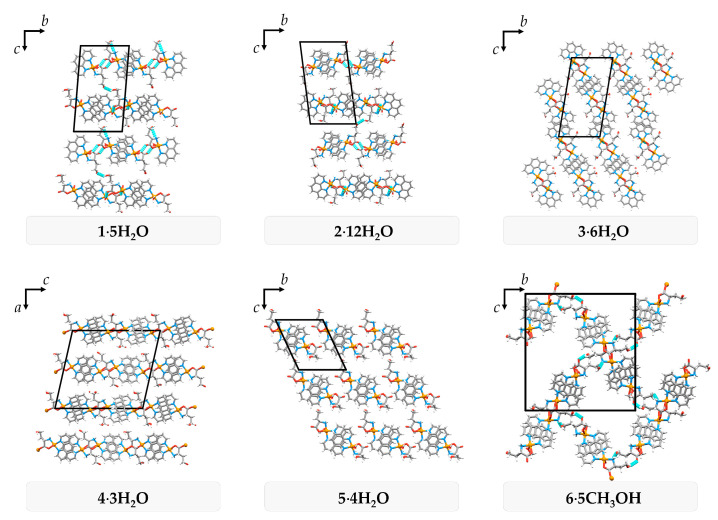
Hydrogen bonds between π-stacked 1D pillars in crystallographic planes perpendicular to the propagation of pillars.

All six compounds form similar packing patterns and supramolecular synthons. The 1,10-phenanthroline ligand has a rigid structure and large flat surface of the aromatic rings. Hence, these compounds tend to assemble through π interactions. Having l-homoserinate ligand on the opposite side of the complex species, aromatic systems form infinite π-stacked 1D pillars. Neighbouring π-stacked pillars in **1∙5H_2_O** and **2∙12H_2_O** form 2D bilayers through O_water_–H∙∙∙O_carboxylate_ and O_hydroxyl_–H∙∙∙O_hydroxyl_ hydrogen bonds, and coordination of a carboxylate oxygen atom to the adjacent complex (only in **2∙12H_2_O**; Figure 4 and Figure S5, Table S5). The **3∙6H_2_O** and **4∙3H_2_O** pillars form 2D monolayers through coordination of a carboxylate oxygen atom to the adjacent complex and O_water_–H∙∙∙O_carboxylate_ hydrogen bonds (only in **3∙6H_2_O**; Figure 4 and Figure S5, Table S6). π-stacked pillars in **5∙4H_2_O** are not directly connected through non-covalent interactions, but only by bridging through the crystallization water molecules and sulfate ions (Figure 4 and Figure S5, Table S7). The solvent **6∙5CH_3_OH** forms 3D framework of π-stacked pillars through the coordination of a carboxylate oxygen atom to the adjacent complex and O_hydroxyl_–H∙∙∙O_carboxylate_ and N–H∙∙∙O_hydroxyl_ hydrogen bonds (Figure 4 and Figure S5, Table S7). Due to numerous hydrogen bond donors and acceptors, all six compounds form a complex 3D hydrogen-bonding network, with preserved synthons O_hydroxyl_–H∙∙∙O_sulfate_, N–H∙∙∙O_sulfate_∙∙∙H–N and O_water/methanol_–H∙∙∙O_carboxylate_ in all compounds (Tables S5–S7).

**Figure 5:**
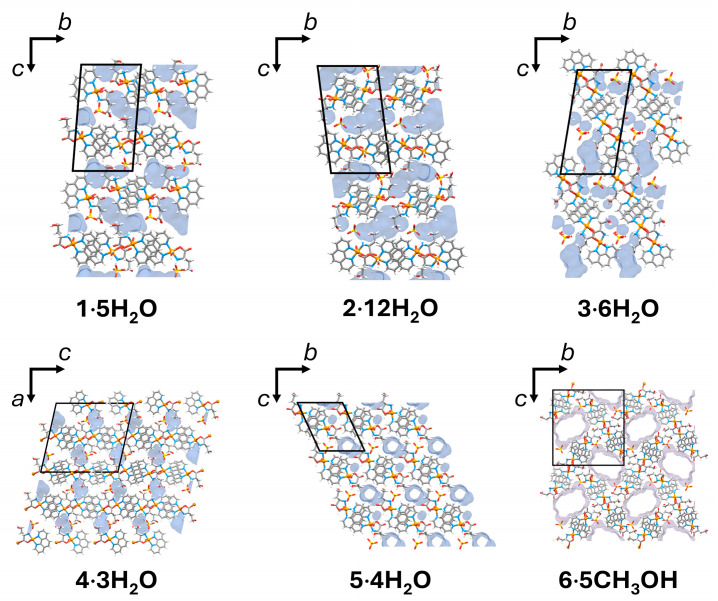
The contact surface of the crystallization water (blue) and methanol (violet) molecules packed in discrete pockets (**1∙5H_2_O**, **2∙12H_2_O**, **3∙6H_2_O**, **4∙3H_2_O**), pockets and 1D channels (**5∙4H_2_O**) and 1D channels (**6∙5CH_3_OH**).

Most of the compounds contain crystallization solvent molecules packed in discrete pockets (**1∙5H_2_O**, **2∙12H_2_O**, **3∙6H_2_O**, **4∙3H_2_O**) with a total volume fraction of 6.4–14.8%. In **5∙4H_2_O**, water molecules are packed both in pockets and 1D channels (volume fraction 11.2%), and **6∙5CH_3_OH** forms large 1D channels occupied by methanol molecules (volume fraction 27.8%) with approximate pore dimensions of 10 × 7 Å^2^ (Figure 5). Large channels and weakly bonded methanol molecules in **6∙5CH_3_OH** are the probable reason for the dynamic disorder of methanol molecules and crystal instability at room temperature outside of the solution. If crystals are soaked in oil, it is possible to keep crystals stable at room temperature.

### 2.3. ESR Spectroscopy

**Figure 6:**
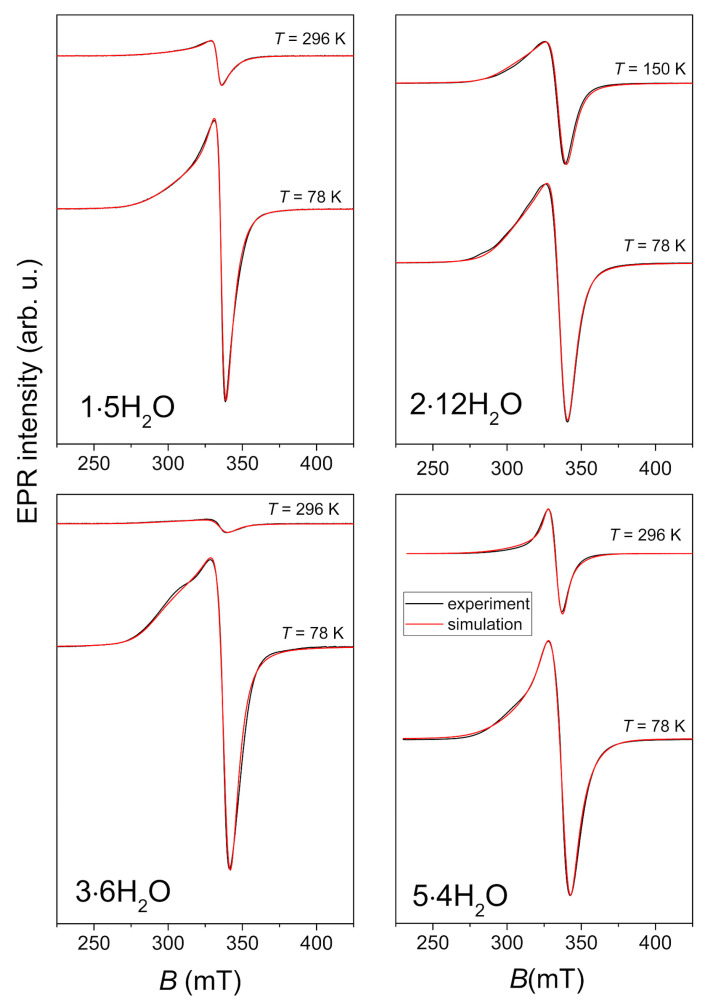
Experimental (black lines) and simulated (red lines) ESR spectra of polycrystalline samples of the investigated complexes. The ESR intensities of the spectra at different temperatures are presented in the real ratios.

Four polycrystalline Cu(II) complexes: **1·5H_2_O**, **2·12H_2_O**, **3·6H_2_O** and **5·4H_2_O** were investigated by X-band electron paramagnetic resonance (EPR) or electron spin resonance (ESR) spectroscopy. Powder diffraction patterns of bulk samples of **1·5H_2_O**, **3·6H_2_O** and **5·4H_2_O** are given in Figures S6–S8. Crystals of **2·12H_2_O** were manually picked and their purity was checked by measuring unit cell parameters. The complex **4·3H_2_O** was synthesized in a low yield and the complex **6·5CH_3_OH** was unstable, therefore their ESR spectra were not recorded. The representative spectra, obtained at a few selected temperatures, are shown in Figure 6. Due to the large amount of solvent, the complex **2·12H_2_O** was wet at room temperature, and therefore its spectrum was recorded at 150 K instead of the room temperature.

The spectral simulations were performed by EasySpin software version number 5.2.36 [34] using the following form of the spin Hamiltonian for copper(II) ions [35,36]:

**H** = *μ*_B_ **B** · **g** ·**S** + **S**·**A**·**I**, (1)

In Equation (1) *μ*_B_ is the Bohr magneton constant, **g** is the **g**-tensor, **B** is the magnetic field vector, **S** and **I** are the electron and nuclear spin operators, respectively, while the hyperfine tensor **A** describes interaction between copper electron and nuclear spins.

The spectra were simulated using the same values of **g**- and **A**-tensor at different temperatures, allowing linewidth of the assumed Lorentzian lineshape to change with temperature. The spin Hamiltonian values, obtained from the simulations, are given in Table 2. The small variations in the local geometry of the Cu(II) coordination can cause distribution of *g_x_*, *g_y_* and *g_z_*-values around some average values. This effect described by **g**_strain_ parameters is also considered in the simulation while these values are presented in Table 2.

The obtained spin Hamiltonian values for copper ions agree with the structural coordination of the ions. Namely, *g*-values *g*_x_ ≈ *g*_y_ < *g*_z_ (as can be seen in Table 2) are expected for the elongated octahedral, square pyramidal or square planar copper geometry [37]. This is in agreement with XRD results that all copper ions in the investigated compounds have elongated square-pyramidal or octahedral coordination. Additionally, the obtained ESR spectra point to the absence of strong exchange interaction between copper centers being in agreement with the fact that the shortest Cu∙∙∙Cu distances in the compounds are more than 5 Å (the shortest distance is 5.157(2) Å in **2∙12H_2_O**).

### 2.4. The Antiproliferative Activities

The antiproliferative activities of **3·6H_2_O** were tested on human cell lines, including HCT116 (colon carcinoma), MCF-7 (breast carcinoma) and H 460 (lung carcinoma), using the MTT test. The tested compound showed pronounced antiproliferative activity toward tested cell lines (Table 3, Figure S9). The *IC*_50_ concentrations are comparable to two known antitumor compounds, etoposide and 5-fluorouracil [38,39]. The activities tested in three tumor cell lines (HTC116, MCF-7 and H460) resulted in *IC*_50_ values in the micromolar range, corresponding to the ones described in the literature [11,40,41].

The compound can be described as a potent cytotoxic agent. However, further mechanistic studies should be performed (DNA binding abilities, ROS generation, induction of apoptosis).

## 3. Materials and Methods

Copper(II) sulfate pentahydrate (Gram-mol, Zagreb, Croatia), copper(II) hydroxide (Alfa Aesar, Ward Hill, MA, USA), l-homoserine (Apollo Scientific, London, UK), anhydrous 1,10-phenanthroline (Apollo Scientific, London, UK) and methanol (Alkaloid, Skopje, North Macedonia) were purchased and used without further purification. Copper(II) sulfate trihydrate, monohydrate and anhydrous copper(II) sulfate were prepared by heating copper(II) sulfate pentahydrate to 60 °C, 120 °C and 220 °C respectively, and the purity was confirmed by powder X-ray diffraction.

Powder X-ray diffraction (PXRD) data were collected on a Malvern Panalytical Aeris diffractometer in a Bragg–Brentano geometry with Cu*K*_α_ radiation (*λ* = 1.54056 Å) equipped with PixCel^1D^ detector. Data collection was taken in the 2*θ* range of 5–40° with a step size 0.022° and measurement of 15.0 s per step. PXRD data were visualized and analyzed using HighscorePlus suite, version 5.2 and DataViewer, version 1.9a [42,43].

The ESR measurements were performed on a Bruker Elexsys 580 FT/CW spectrometer in the temperature range from the room down to liquid nitrogen temperature. The frequency of microwaves was around 9.7 GHz with a magnetic field modulation amplitude of 0.5 mT and modulation frequency of 100 kHz.

### 3.1. Single-Crystal X-ray Diffraction (SCXRD)

#### 3.1.1. Data Collection and Struture Refinement

Single-crystal X-ray diffraction data of **2∙12H_2_O**, **4∙3H_2_O** and **6∙5CH_3_OH** were collected at the Elettra Sincrotrone Trieste facility on XRD1 and XRD2 beamlines at 100 K with synchrotron radiation wavelength set to 0.70000 Å. X-ray diffraction data for **3∙6H_2_O** were collected at 150 K on an Oxford Diffraction Xcalibur3 diffractometer equipped with a CCD detector using Mo*K*_α_ radiation (*λ* = 0.71073 Å). X-ray diffraction data for **1∙5H_2_O** and **5∙4H_2_O** were collected on a Rigaku XtaLAB Synergy-S diffractometer equipped with a HyPix 6000HE detector using Cu*K*_α_ radiation (*λ* = 1.54056 Å) at 170 K. The diffraction data of all compounds were processed using CrysalisPro software, version 171.42.63a [44]. All crystal structures were solved using SHELXS, version 2013/1 [45] and refined using SHELXL, version 2018/3 [46] programs incorporated within the WinGX software package, version 2023.1 [47]. Structures were visualized by the Mercury program, version 2024.1.0 [48]. Geometrical parameters were calculated with PLATON, version v1.17 [49]. All non-hydrogen atoms except one carbon and one oxygen atom of the methanol molecule in **6∙5CH_3_OH** were refined anisotropically. Most of the hydrogen atoms were placed at calculated positions according to the idealized geometry of the respective functional group. Hydrogen atoms in water molecules were found in the difference Fourier map and restrained by DFIX to O–H distance 0.85(1) Å and by DANG to H∙∙∙H distance 1.39(2) Å. Some of the hydrogen atom positions of water molecules were fixed due to disorder or poor-quality data. Three methanol molecules in **6∙5CH_3_OH** were not modelled with hydrogen atoms due to the high dynamic disorder of those molecules. Atoms and molecules in static disorder were modelled as two parts (one sulfate ion in **2∙12H_2_O**, one sulfate ion, one water molecule and OH group of one l-homoserinate ligand in **3∙6H_2_O**, OH group of one l-homoserinate ligand and two crystallization water molecules in **4∙3H_2_O** and one crystallization methanol molecule in **6∙5CH_3_OH**) with a total occupation factor constrained to 1, with the respective occupation factor of each disordered part refined as a free variable.

#### 3.1.2. Crystal Structure Data

Crystal data for **1∙5H_2_O**, C_32_H_46_Cu_2_N_6_O_17_S (*M* = 945.89 g/mol): triclinic, space group *P*1 (no. 1), *a* = 7.0350(2) Å, *b* = 12.4003(3) Å, *c* = 22.1264(5) Å, α = 94.104(2)°, *β* = 95.416(2)°, *γ* = 95.024(2)°, *V* = 1908.01(8) Å^3^, *Z* = 2, *T* = 170 K, *μ*(Cu*K*_α_) = 2.623 mm^−1^, *D*_calc_ = 1.646 g/cm^3^, 49,640 reflections measured (7.2° ≤ 2*Θ* ≤ 140.0°), 13,470 unique (*R*_int_ = 0.101, *R*_sigma_ = 0.0652) which were used in all calculations. The final *R*_1_ was 0.0775 (*I* > 2*σ*(*I*)) and w*R*_2_ was 0.2462 (all data).

Crystal data for **2∙12H_2_O**, C_32_H_48_Cu_2_N_6_O_18_S (*M* = 1927.80 g/mol): triclinic, space group *P*1 (no. 1), *a* = 7.0513(1) Å, *b* = 12.4674(3) Å, *c* = 22.4771(4) Å, α = 82.743(2)°, *β* = 89.100(1)°, *γ* = 84.595(2)°, *V* = 1951.43(7) Å^3^, *Z* = 2, *T* = 100 K, *μ*(sync) = 1.178 mm^−1^, *D*_calc_ = 1.640 g/cm^3^, 37,926 reflections measured (3.2° ≤ 2*Θ* ≤ 60.0°), 21,204 unique (*R*_int_ = 0.034, *R*_sigma_ = 0.0409) which were used in all calculations. The final *R*_1_ was 0.0296 (*I* > 2*σ*(*I*)) and w*R*_2_ was 0.0819 (all data).

Crystal data for **3∙6H_2_O**, C_32_H_46_Cu_2_N_6_O_17_S (*M* = 1891.77 g/mol): triclinic, space group *P*1 (no. 1), *a* = 6.9912(2) Å, *b* = 11.8677(3) Å, *c* = 23.1195(5) Å, α = 99.174(2)°, *β* = 93.024(2)°, *γ* = 92.145(2)°, *V* = 1888.99(8) Å^3^, *Z* = 1, *T* = 150 K, *μ*(Mo*K*_α_) = 1.266 mm^−1^, *D*_calc_ = 1.663 g/cm^3^, 34,688 reflections measured (8.4° ≤ 2*Θ* ≤ 56.0°), 18,148 unique (*R*_int_ = 0.026, *R*_sigma_ = 0.0401) which were used in all calculations. The final *R*_1_ was 0.0318 (*I* > 2*σ*(*I*)) and w*R*_2_ was 0.0742 (all data).

Crystal data for **4∙3H_2_O**, C_32_H_38_Cu_2_N_6_O_13_S (*M* = 873.82 g/mol): monoclinic, space group *P2*_1_ (no. 4), *a* = 21.2145(5) Å, *b* = 7.0688(2) Å, *c* = 23.2039(5) Å, *β* = 102.683(2)°, *V* = 3394.78(15) Å^3^, *Z* = 4, *T* = 100 K, *μ*(sync) = 1.276 mm^−1^, *D*_calc_ = 1.710 g/cm^3^, 34,967 reflections measured (3.6° ≤ 2*Θ* ≤ 52.0°), 13,807 unique (*R*_int_ = 0.050, *R*_sigma_ = 0.0419) which were used in all calculations. The final *R*_1_ was 0.0691 (*I* > 2*σ*(*I*)) and w*R*_2_ was 0.2365 (all data).

Crystal data for **5∙4H_2_O**, C_33_H_46_Cu_2_N_6_O_16_S (*M* = 941.90 g/mol): triclinic, space group *P*1 (no. 1), *a* = 7.0634(2) Å, *b* = 11.7650(3) Å, *c* = 13.3013(4) Å, α = 65.858(3)°, *β* = 89.472(3)°, *γ* = 75.321(3)°, *V* = 970.15(6) Å^3^, *Z* = 1, *T* = 170 K, *μ*(Cu*K*_α_) = 2.557 mm^−1^, *D*_calc_ = 1.612 g/cm^3^, 49,945 reflections measured (7.4° ≤ 2*Θ* ≤ 162.0°), 7851 unique (*R*_int_ = 0.041, *R*_sigma_ = 0.0180) which were used in all calculations. The final *R*_1_ was 0.0671 (*I* > 2*σ*(*I*)) and w*R*_2_ was 0.1730 (all data).

Crystal data for **6∙5CH_3_OH**, C_38_H_44_Cu_2_N_6_O_16_S (*M* = 999.93 g/mol; C_38_H_56_Cu_2_N_6_O_16_S, *M* = 1012.26 with missing hydrogen atoms): orthorhombic, space group *P*2_1_2_1_2_1_ (no. 19), *a* = 7.2312(1) Å, *b* = 23.8780(2) Å, *c* = 25.3667(2) Å, *V* = 4379.98(8) Å^3^, *Z* = 4, *T* = 100 K, *μ*(sync) = 1.053 mm^−1^, *D*_calc_ = 1.516 g/cm^3^, 78,619 reflections measured (3.2° ≤ 2*Θ* ≤ 60.0°), 13,318 unique (*R*_int_ = 0.057, *R*_sigma_ = 0.0274) which were used in all calculations. The final *R*_1_ was 0.0402 (*I* > 2*σ*(*I*)) and w*R*_2_ was 0.1134 (all data).

### 3.2. Synthetic Procedures

#### 3.2.1. General Procedure for Preparation of Compounds

Anhydrous copper(II) sulfate or a hydrate of copper(II) sulfate (monohydrate, trihydrate, pentahydrate), copper(II) hydroxide, l-homoserine and 1,10-phenanthroline were placed into a container in a 1:1:2:2 molar ratio and the solvent (water, methanol or a mixture of water and methanol) was added. The mixture was heated for 15 min at the boiling temperature of a solution. Solutions were filtered and left to evaporate at room temperature. Crystals were obtained after a few days or weeks. The scheme of synthetic procedures is shown in Figure 1. Detailed synthetic procedures are given in the Supporting Materials.

#### 3.2.2. Recrystallization **6∙5CH_3_OH** → **3∙6H_2_O**

A few drops of a methanolic saturated solution of **6∙5CH_3_OH** containing crystals were placed on a glass surface. Changes were observed during solvent evaporation and shown in Video S1 and Figure S1 in the Supporting Materials. The composition of crystals was checked by the SCXRD.

### 3.3. Biological Assays

HCT116, H460 and MCF-7 cells were cultured as monolayers and maintained in Dulbecco’s modified Eagle medium (DMEM), supplemented with 10% fetal bovine serum (FBS), 2 mM l-glutamine, 100 U/mL penicillin and 100 μg/mL streptomycin in a humidified atmosphere with 5% CO_2_ at 37 °C. The Supporting Materials contain information on proliferation assays and *IC*_50_ calculation.

## 4. Conclusions

We synthesized and structurally characterized six novel solvates of copper(II) compounds with l-homoserinate and 1,10-phenanthroline. This is only the second structural report of a coordination compound with l-homoserinate. In most synthetic procedures, a mixture of compounds was obtained due to the high dependency of the crystallization process on ever-changing conditions. We demonstrated that **6∙5CH_3_OH**, which is stable within the mother liquor, can dissolve during evaporation, resulting in **3∙6H_2_O**. Compounds **6∙5CH_3_OH** and **3∙6H_2_O** are the easiest to reproduce, although **6∙5CH_3_OH** is unstable outside of solution at room temperature. Structurally, all prepared compounds form square-pyramidal or octahedral coordination compounds, where l-homoserinate and 1,10-phenanthroline are coordinated in the equatorial plane. That type of coordination is responsible for described similarities in crystal packing, increasing the predictability of some non-covalent interactions, which can be used to design novel supramolecular or polymeric coordination compounds. We also plan to use these compounds as model compounds to research coordination polymers of higher dimensionality. All six compounds are solvates, where crystallization water or methanol molecules occupy pockets, 1D channels or both. The largest 1D channels are in **6∙5CH_3_OH** with approximate dimensions of 10 × 7 Å^2^. ESR spectroscopy revealed spin Hamiltonian parameters for copper ions in four compounds. The *g*-values obtained from the spectral simulation points to copper coordination geometry, in agreement with structural analysis. No significant exchange coupling between copper spins was observed. **3∙6H_2_O** showed micromolar antiproliferative activity towards all three tested cell lines, HCT116, MCF-7 and H 460, and are comparable to copper(II) coordination compounds with 1,10-phenanthroline and l-serine, and to etoposide and 5-fluorouracil, well-known antitumor compounds, making them promising antitumor agents.

## Figures and Tables

**Table 1:** Types of complexes in the crystal structures of compounds.

Compound	Types of Complex Species
**1∙5H_2_O**	Type 1
**2∙12H_2_O**	Type 1 and 2
**3∙6H_2_O**	Type 2 and 3
**4∙3H_2_O**	Type 3
**5∙4H_2_O**	Type 1
**6∙5CH_3_OH**	Type 1 and 3

**Table 2:** The spin Hamiltonian values obtained from the spectral simulations, together with the parameter used in the simulations: **g**_strain_ and linewidths l_w_.

Complex	g = [*g_x_ g_y_ g_z_*]	g_strain_	A (MHz)	*l_w_* (mT)	*T* (K)
**1·5H_2_O**	[2.06 2.06 2.23]	[0.0 0.0 0.35] [0.0 0.26 0.28]	[0 0 146]	3.9 4.7	296 78
**2·12H_2_O**	[2.05 2.08 2.22]	[0.0 0.0 0.15] [0.0 0.0 0.15]	[0 0 246]	7.5 7.5	150 78
**3·6H_2_O**	[2.05 2.06 2.27]	[0.0 0.0 0.15] [0.0 0.0 0.20]	[0 0 270]	9.5 8	296 78
**5·4H_2_O**	[2.04 2.08 2.18]	[0.04 0.04 0.38] [0.03 0.05 0.36]	[0 0 136]	0.2 3.8	296 78

**Table 3:** *IC*_50_ values (in µM).

Compound	*IC*_50_^a^/10^−6^ mol dm^−3^		
	Cell Lines		
	HCT116	MCF-7	H 460
**3·6H_2_O**	1.5 ± 0.3	1.7 ± 0.02	2.13 ± 0.17
**[Cu(l-ser)(H_2_O)(phen)]_2_SO_4_∙6H_2_O**	- ^b^	2 ± 0.08 ^c^	2 ± 0.2 ^c^
**etoposide**	5 ± 2 ^d, e^	1 ± 0.7 ^d, e^	0.1 ± 0.04 ^d, e^
**5-fluorouracil**	4 ± 1 ^e^	14 ± 0.3 ^e^	3 ± 0.3 ^e^

^a^ *IC*_50_—concentration that causes 50% growth inhibition; ^b^ not measured; ^c^ ref. [11]; ^d^ ref. [38]; ^e^ ref. [39].

## Data Availability

The original contributions presented in the study are included in the article/Supplementary Material, further inquiries can be directed to the corresponding author.

## Supplementary Materials

The following supporting information can be downloaded at: https://www.mdpi.com/article/10.3390/molecules29235621/s1, Table S1: Crystallographic data for compounds **1∙5H_2_O**, **2·12H_2_O** and **3·6H_2_O**; Table S2: Crystallographic data for compounds **4·3H_2_O**, **5·4H_2_O** and **6·5CH_3_OH**; Table S3: Distances ([Å]) within the polyhedra of copper coordination spheres in the crystal structures of **1·5H_2_O**, **2·12H_2_O**, **3·6H_2_O, 4·3H_2_O**, **5·4H_2_O** and **6·5CH_3_OH**; Table S4: *τ*_5_-parameters in complex cations with pentacoordinated copper atoms; Table S5: Selected hydrogen bonds for compounds **1·5H_2_O** and **2·12H_2_O**; Table S6: Selected hydrogen bonds for compounds **3·6H_2_O** and **4·3H_2_O**; Table S7: Selected hydrogen bonds for compounds **5·4H_2_O** and **6·5CH_3_OH**; Figure S1: Screenshots from Video S1 showing transformation **6·5CH_3_OH→3·6H_2_O** at time frames: (a) 60 s with crystals of **6∙5CH_3_OH**; (b) 120 s with crystals of **6∙5CH_3_OH**; (c) 160 s with crystals of **6∙5CH_3_OH**; (d) 200 s; (e) 280 s with crystals of **3∙6H_2_O**; (f) 360 s with crystals **3∙6H_2_O**. Figure S2: Asymmetric unit of **1∙5H_2_O** and **2∙12H_2_O**. Crystallization water molecules and sulfate are omitted for clarity.; Figure S3: Asymmetric unit of **3∙6H_2_O** and **4∙3H_2_O**. Crystallization water molecules and sulfate are omitted for clarity.; Figure S4: Asymmetric unit of **5∙4H_2_O** and **6∙5CH_3_OH**. Crystallization water/methanol molecules and sulfate are omitted for clarity.; Figure S5: Hydrogen bonds and coordinative bridging between π-stacked 1D pillars.; Figure S6: Experimental (blue) PXRD pattern obtained from synthesis and PXRD patternt calculated from crystal structure of **1∙5H_2_O** (red); Figure S7: Experimental (blue) PXRD pattern obtained from synthesis and PXRD patternt calculated from crystal structure of **3∙6H_2_O** (red); Figure S8: Experimental (blue) PXRD pattern obtained from synthesis and PXRD patternt calculated from crystal structure of **5∙4H_2_O** (red); Figure S9: Concentration–response profiles for **3·6H2O** tested in vitro on HCT116, MCF-7 and H 460 cell lines; Video S1: Transformation **6·5CH_3_OH→3·6H_2_O** by evaporation. Video was accelerated 10 times. CCDC 2392866-2392871 contains the supplementary crystallographic data for this paper. These data can be obtained free of charge via https://www.ccdc.cam.ac.uk/structures/ (accessed on 23 October 2024) or from the CCDC, 12 Union Road, Cambridge CB2 1EZ, UK; Fax: +44 1223 336033; E-mail: deposit@ccdc.cam.ac.uk.
