# Supplementary material for: Solvatomorphic Diversity in Coordination Compounds of Copper(II) with l-Homoserine and 1,10-Phenanthroline: Syntheses, Crystal Structures and ESR Study
Source: Molecules. 2024 Nov 27;29(23):5621. doi: 10.3390/molecules29235621 (PMC11643913; doi:10.3390/molecules29235621)
Supplement: Supplementary file 1 [file molecules-29-05621-s001.zip › molecules-3304768-supplementary.pdf]

Supplementary Materials

Solvatomorphic diversity in coordination compounds of copper(II) with L-homoserine and 1,10-phenanthroline: syntheses, crystal structures and ESR study

Darko Vušak,[1] Marta Šimunović Letić,[1] Marina Tašner,[1] Dubravka Matković-Čalogović,[1] Jurica Jurec,[2] Dijana Žilić,[2] Biserka Prugovečki[1*]

[1] Department of Chemistry, Faculty of Science, University of Zagreb, Horvatovac 102A, HR-10000 Zagreb, Croatia; dvusak@chem.pmf.hr, marta.simunovic12@gmail.com, mtasner@chem.pmf.hr, dubravka@chem.pmf.hr, biserka@chem.pmf.hr
[2] Laboratory for Magnetic Resonances, Division of Physical Chemistry, Ruđer Bošković Institute, Bijenička cesta 54, HR-10000 Zagreb, Croatia; Jurica.Jurec@irb.hr, dzilic@irb.hr
* Correspondence: biserka@chem.pmf.hr (BP)

Syntheses

*Synthesis of [Cu(μ-L-hser)(H_2O)(phen)][Cu(L-hser)(H_2O)(phen)]$_3$(SO_4)$_2$·12H_2O (**2·12H$_2$O**) and {[Cu(μ-Hser)(H_2O)(phen)][Cu(μ-Hser)(phen)]SO_4·6H_2O}$_n$ (**3·6H$_2$O**).*

Copper(II) sulfate pentahydrate (62.4 mg, 0.25 mmol), copper(II) hydroxide (24.4 mg, 0.25 mmol), L-homoserine (59.6 mg, 0.50 mmol) and 1,10-phenanthroline (90.1 mg, 0.50 mmol) were placed into a glass container and 10 mL of water was added. A mixture was heated at boiling point for 15 minutes, filtered, and the filtrate was left at room temperature to evaporate. After a few days, blue crystals of **2·12H$_2$O** or **3·6H$_2$O** crystallized from the solution. In some syntheses, **2·12H$_2$O**, and in some syntheses, **3·6H$_2$O** were obtained. After several repeated experiments, we observed that **3·6H$_2$O** is formed more often. Probably small differences in evaporation rate can affect the final product, sometimes giving different products, or a mixture of products. Crystals of **2·12H$_2$O** or **3·6H$_2$O** are stable outside the solution and are of good quality for the single-crystal X-ray diffraction experiment. Yield for syntheses, where **3·6H$_2$O** was the sole product is 72–77 %.

*Synthesis of {[Cu(μ-Hser)(H_2O)(phen)][Cu(μ-Hser)(phen)]SO_4·6H_2O}$_n$ (**3·6H$_2$O**) and [Cu(Hser)(H_2O)(phen)]$_2$$SO_4$·5$H_2O$ (**1·5H$_2$O**).*

Copper(II) sulfate pentahydrate (62.4 mg, 0.25 mmol), copper(II) hydroxide (24.4 mg, 0.25 mmol), L-homoserine (59.6 mg, 0.50 mmol) and 1,10-phenanthroline (90.1 mg, 0.50 mmol) were placed into a glass container and 10 mL of a mixture of water and methanol (3:7, *v*/*v*) was added. A mixture was heated at boiling point for 15 minutes, filtered and the filtrate was left at room temperature to evaporate. After a few days, blue needles of **3·6H$_2$O** form on the edges of a crystallizing dish. If the solution is dropped on a glass surface and let to evaporate, light blue needles of **1·5H$_2$O** form on the edges of a drop. Crystals of **1·5H$_2$O** are stable outside of solution, and the crystals are quality enough for the single-crystal X-ray diffraction experiment. If the same reactants and a mixture of water and methanol (7:3, *v*/*v*) was used, only **3·6H$_2$O** crystallized from solution.

*Synthesis of $[Cu(Hser)(H_2O)(phen)]_2SO_4{\cdot}5H_2O$ (**1·5H2O**), $\{[Cu(\mu\text{-}Hser)(H_2O)(phen)][Cu(\mu\text{-}Hser)(phen)]SO_4{\cdot}6H_2O\}_n$ (**3·6H2O**), and $\{[Cu(\mu\text{-}Hser)(phen)]_2SO_4{\cdot}3H_2O\}_n$ (**4·3H2O**).*

Copper(II) sulfate pentahydrate (62.4 mg, 0.25 mmol), copper(II) hydroxide (24.4 mg, 0.25 mmol), L-homoserine (59.6 mg, 0.50 mmol) and 1,10-phenanthroline (90.1 mg, 0.50 mmol) were placed into a glass container and 10 mL of a mixture of water and methanol (1:1, *v*/*v*) was added. A mixture was heated at boiling point for 15 minutes, filtered, and the filtrate was left at room temperature to evaporate. After a few days, a mixture of light blue needles of **1·5H2O** or blue prismatic crystals of **4·3H2O** (few crystals) or blue needles of **3·6H2O** were formed. Crystals of **1·5H2O** are stable outside the solution, while the crystals of **4·3H2O** are unstable outside the solution. The crystals were tiny and started to decompose after more prolonged exposure to X-ray irradiation, so we could not collect high-quality single-crystal X-ray diffraction data

*Synthesis of $[Cu(Hser)(H_2O)(phen)][Cu(Hser)(CH_3OH)(phen)]SO_4{\cdot}4H_2O$ (**5·4H2O**) and/or $\{[Cu(\mu\text{-}Hser)(phen)][Cu(Hser)(CH_3OH)(phen)]SO_4{\cdot}5CH_3OH\}_n$ (**6·5CH3OH**).*

Copper(II) sulfate pentahydrate (62.4 mg, 0.25 mmol), copper(II) hydroxide (24.4 mg, 0.25 mmol), L-homoserine (59.6 mg, 0.50 mmol) and 1,10-phenanthroline (90.1 mg, 0.50 mmol) were placed into a glass container and 10 mL of methanol was added. A mixture was heated at boiling point for 15 minutes, filtered and the filtrate was left at room temperature in a closed container. After a few days, blue crystals of **5·4H2O** or **6·5CH3OH** were formed. After several repeated experiments, we observed that **6·5CH3OH** formed more often. Crystals of **5·4H2O** are stable outside of the solution, while crystals of **6·5CH3OH** decompose immediately when taken out of the solution. The crystals of both compounds were quality enough for the X-ray single-crystal diffraction experiment.

*Synthesis of $\{[Cu(\mu\text{-}Hser)(phen)][Cu(Hser)(CH_3OH)(phen)]SO_4{\cdot}5CH_3OH\}_n$ (**6·5CH3OH**).*

Copper(II) sulfate trihydrate (53.4 mg, 0.25 mmol), copper(II) hydroxide (24.4 mg, 0.25 mmol), L-homoserine (59.6 mg, 0.50 mmol) and 1,10-phenanthroline (90.1 mg, 0.50 mmol) were placed into a glass container and 10 mL of methanol was added. A mixture was heated at boiling point for 15 minutes, filtered and the filtrate was left at room temperature in a closed container. After a few days, blue crystals of **6·5CH3OH** in a mixture with light blue crystals of $[Cu(SO_4)(phen)_2]{\cdot}CH_3OH$ (refcode in CSD: MUNHIO) were formed. The same result is obtained if copper(II) sulfate monohydrate (44.4 mg, 0.25 mmol) or anhydrous copper(II) sulfate (39.9 mg, 0.25 mmol) were used instead of copper(II) sulfate trihydrate.

a) b) c)

d) e) f)

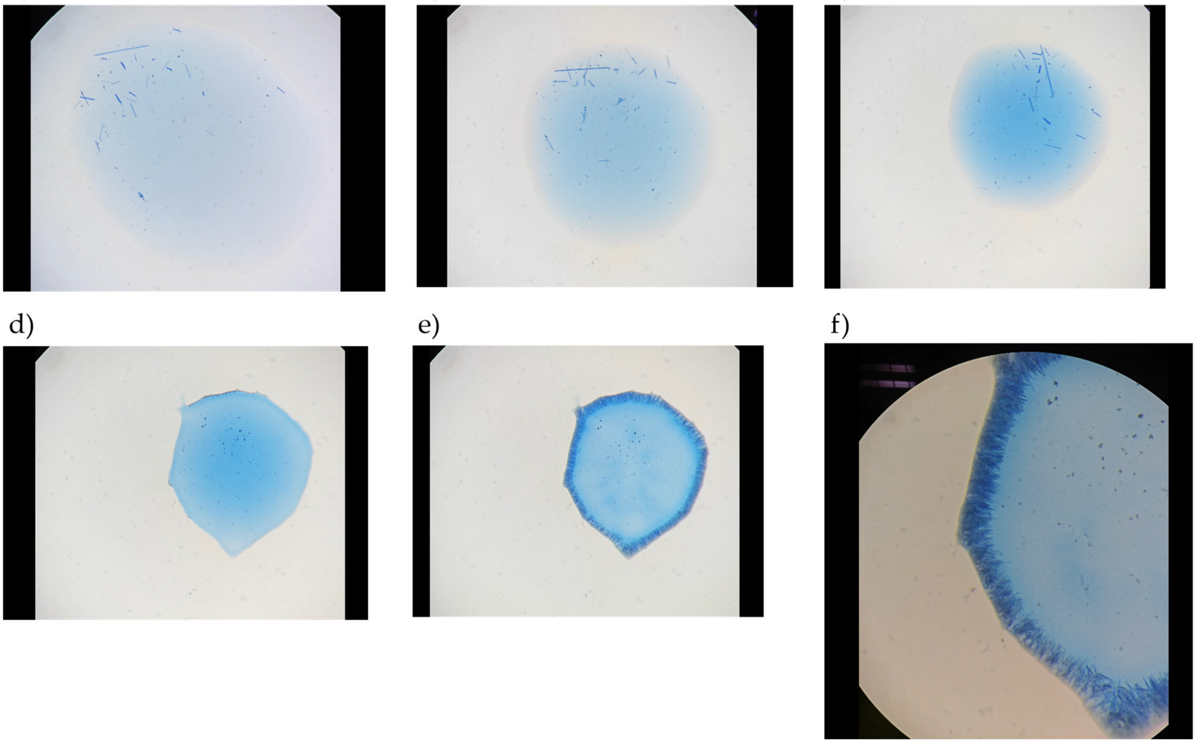

Figure S1. Screenshots from Video 1 showing transformation **6·5CH_3OH→3·6H_2O** at different time frames after statrting the experiment: a) 60 s with crystals of **6·5CH_3OH**; b) 120 s with crystals of **6·5CH_3OH**; c) 160 s with crystals of **6·5CH_3OH**; d) 200 s; e) 280 s with crystals of **3·6H_2O**; f) 360 s with crystals **3·6H_2O**.

Crystal structures

Table S1. Crystallographic data for compounds **1·5H2O**, **2·12H2O** and **3·6H2O.**

	**1·5H2O**	**2·12H2O**	**3·6H2O**
Chemical formula	$C_{32}H_{46}Cu_2N_6O_{17}S$	$C_{32}H_{48}Cu_2N_6O_{18}S$	$C_{32}H_{46}Cu_2N_6O_{17}S$
Molecular weight [g mol^{-1}]	945.89	1927.80	1891.77
Radiation [Å]	1.54184	sync. 0.70000	0.71073
T [K]	170	100	150
Crystal colour and shape	light blue, needle	blue, needle	blue, needle
crystal size [mm^3]	0.19×0.03×0.02	0.10×0.02×0.01	0.63×0.22×0.08
Crystal system	triclinic	triclinic	triclinic
Space group	$P1$	$P1$	$P1$
a [Å]	7.0350(2)	7.0513(1)	6.9912(2)
b [Å]	12.4003(3)	12.4674(3)	11.8677(3)
c [Å]	22.1264(5)	22.4771(4)	23.1195(5)
α [°]	94.104(2)	82.743(2)	99.174(2)
β [°]	95.416(2)	89.100(1)	93.024(2)
γ [°]	95.024(2)	84.595(2)	92.145(2)
V [Å^3]	1908.01(8)	1951.43(7)	1888.99(8)
Z	2	2	1
ρ [g cm^{-3}]	1.646	1.640	1.663
μ [mm^{-1}]	2.623	1.178	1.266
θ range [°]	3.6, 70.0	1.6, 30.0	4.2 ,28.0
Independent reflections (R_{int})	0.101	0.034	0.026
Observed reflections ($I > 2\ \sigma\ (I)$)	10403	20597	17148
Number of parameters	1103	1175	1162
R_1 (observed reflections)	0.0775	0.0296	0.0318
wR_2 (all data)	0.2462	0.0819	0.0742
S	1.12	1.07	1.03
max/min residual electron density [e Å^{-3}]	0.74,-1.14	0.46,-0.85	0.65,-0.48
CCDC no.	2392867	2392866	2392869

Table S2. Crystallographic data for compounds 4·**3H$_2$O**, 5·**4H$_2$O** and **6·5CH$_3$OH**.

	**4·3H$_2$O**	**5·4H$_2$O**	**6·5CH$_3$OH**
Chemical formula	$C_{32}H_{38}Cu_2N_6O_{13}S$	$C_{33}H_{46}Cu_2N_6O_{16}S$	$C_{38}H_{44}Cu_2N_6O_{16}S$[a]
Molecular weight [g mol^{-1}]	873.82	941.90	999.93[a]
Radiation [Å]	sync. 0.70000	1.54184	sync. 0.70000
T [K]	100	170(2)	100
Crystal colour and shape	blue, plate	blue, plate	blue, plate
crystal size [mm^3]	0.18×0.04×0.03	0.29×0.12×0.06	0.33×0.10×0.08
Crystal system	monoclinic	triclinic	orthorhombic
Space group	$P2_1$	$P1$	$P2_12_12_1$
a [Å]	21.2145(5)	7.0634(2)	7.2312(1)
b [Å]	7.0688(2)	11.7650(3)	23.8780(2)
c [Å]	23.2039(5)	13.3013(4)	25.3667(2)
α [°]	90	65.858(3)	90
β [°]	102.683(2)	89.472(3)	90
γ [°]	90	75.321(3)	90
V [Å^3]	873.82	970.15(6)	4379.98(8)
Z	4	1	4
ρ [g cm^{-3}]	1.710	1.612	1.516
μ [mm^{-1}]	1.276	2.557	1.053
θ range [°]	1.8, 26.0	3.7, 81.0	1.6, 30.0
Independent reflections (R_{int})	0.050	0.041	0.057
Observed reflections ($I > 2\ \sigma\ (I)$)	12138	7799	12780
Number of parameters	1009	669	598
R_1 (observed reflections)	0.0691	0.0671	0.0402
wR_2 (all data)	0.2365	0.1730	0.1134
S	1.08	1.11	1.03
max/min residual electron density [e Å^{-3}]	1.30, -1.33	0.43, -0.75	0.85, -0.75
CCDC no.	2392871	2392870	2392868

[a] Hydrogen atoms were not found for all atoms in disordered methanol molecules. If missing hydrogen atoms are considered, formula of a compound is $C_{38}H_{56}Cu_2N_6O_{16}S$ and molecular weight is 1012.26.

Table S3. Distances ([Å]) within the polyhedra of copper coordination spheres in the crystal structures of **1·5H_2O**, **2·12H_2O**, **3·6H_2O, 4·3H_2O**, **5·4H_2O** and **6·5CH_3OH.**

Bond lengths[Å]

	**1·5H_2O**	**2·12H_2O**	**3·6H_2O**	**4·3H_2O**	**5·4H_2O**	**6·5CH_3OH**
Cu1-O11	1.950(10)	1.965(2)	1.948(3)	1.958(9)	1.943(5)	1.938(3)
Cu1-O12	-	-	-	2.363(14)[a]	-	-
Cu1-O42	-	2.855(4)	-	-	-	-
Cu2-O21	1.953(9)	1.966(3)	-	1.932(8)	1.945(6)	1.945(2)
Cu2-O22	-	-	1.951(3)	2.337(14)[b]	-	2.313(2)[e]
Cu2-O42	-	-	2.547(3)[b]	-		-
Cu3-O12	-	-	2.365(3)	-		-
Cu3-O31	1.940(10)	1.944(3)	1.939(3)	1.951(9)	-	
Cu3-O32	-	-	-	2.387(14)[c]	-	-
Cu4-O21	-	-	2.346(3)	-		-
Cu4-O41	1.925(10)	1.942(2)	1.933(3)	1.952(8)	-	-
Cu4-O42	-	-	-	2.307(13)[d]	-	-
Cu1-O13	2.278(10)	2.313(4)	2.353(3)	-	2.214(6)	-
Cu2-O23	2.219(11)	2.234(4)	2.496(3)	-	-	-
Cu3-O33	2.268(11)	2.256(4)	-	-	-	-
Cu4-O43	2.273(11)	2.294(4)	-	-	-	-
Cu1-O13M	-	-	-	-	-	2.216(3)
Cu2-O23M	-	-	-	-	2.228(5)	-
Cu1-N1	2.020(13)	2.000(3)	2.005(3)	1.982(8)	1.980(5)	1.996(3)
Cu2-N2	1.994(13)	1.987(3)	1.985(3)	1.992(8)	1.994(5)	1.986(2)
Cu3-N3	1.991(13)	2.001(3)	1.982(3)	1.967(8)	-	-
Cu4-N4	1.992(13)	1.998(3)	2.005(3)	1.975(8)	-	-
Cu1-N11	2.025(11)	2.001(2)	2.012(3)	2.008(7)	2.015(6)	2.013(3)
Cu1-N110	2.028(11)	2.018(3)	2.012(3)	2.021(8)	2.021(6)	2.006(3)
Cu2-N21	2.010(13)	1.998(2)	2.000(3)	2.012(7)	2.009(5)	2.000(2)
Cu2-N210	2.016(11)	2.020(3)	2.005(3)	2.005(9)	2.010(6)	2.017(3)
Cu3-N31	2.007(13)	2.013(3)	2.001(4)	2.007(8)	-	-
Cu3-N310	2.007(11)	2.010(3)	2.019(3)	2.025(8)	-	-
Cu4-N41	1.990(13)	2.005(3)	2.011(3)	2.011(8)	-	-
Cu4-N410	2.029(12)	2.019(3)	2.021(3)	2.035(8)	-	-

[a]1-x,-1/2+y,-z; [b]1-x,1/2+y,1-z; [c]2-x,-1/2+y,-z; [d]2-x,1/2+y,1-z ; [e]1/2+x,3/2-y,1-z

Table S4. τ_5-parameters in complex cations with pentacoordinated copper atoms.

Bond lengths[Å]

Compound	Copper atom	τ_5
**1·5H$_2$O**	Cu1	0.190
	Cu2	0.190
	Cu3	0.121
	Cu4	0.006
**2·12H$_2$O**	Cu2	0.188
	Cu3	0.198
	Cu4	0.029
**3·6H$_2$O**	Cu1	0.046
	Cu3	0.083
	Cu4	0.150
**4·3H$_2$O**	Cu1	0.102
	Cu2	0.163
	Cu3	0.023
	Cu4	0.065
**5·4H$_2$O**	Cu1	0.193
	Cu2	0.06
**6·5CH$_3$OH**	Cu1	0.132
	Cu2	0.053

Table S5. Selected hydrogen bonds for compounds **1·5H$_2$O** and **2·12H$_2$O.**

**Compound**	**D–H···A**	***d*(D–H···A) / Å**	**∠(D–H···A) / °**
**1·5H$_2$O**	O4D–H4D···O2D[a]	2.612(15)	139
	O13–H13B···O22[b]	2.719(14)	179
	O23–H23A···O12[c]	2.809(15)	159(10)
	O33–H33B···O42[d]	2.715(17)	175
	O43–H43B···O32[e]	2.763(16)	161(10)
	O2D–H2D···O14S[f]	2.654(14)	170
	O3D–H3D···O21S[g]	2.70(2)	157
	N1–H1A···O24S[h]	2.939(17)	163
	N2–H2A···O11S[c]	3.09(3)	172
	N3–H3A···O22S[g]	3.03(2)	161
	N3–H3B···O23S[h]	3.13(2)	161
**2·12H$_2$O**	O4D–H4D···O2D[j]	2.622(3)	128
	O13–H13B···O42[i]	2.781(4)	160(3)
	O23–H23A···O32[c]	2.762(3)	164(3)
	O33–H33B···O22[g]	2.708(3)	151(3)
	O43–H43B···O12[e]	2.733(4)	168
	O1D–H1D···O23S[e]	3.056(4)	140
	O2D–H2D···O11S[k]	2.649(5)	170
	O3D–H3D···O22S[g]	2.601(3)	166
	N1–H1B···O23S[e]	3.056(4)	140
	N2–H2B···O14S[g]	2.772(6)	142
	N2–H2B···O16S[g]	2.902(7)	174
	N2–H2C···O15S[k]	3.353(8)	144
	N3–H3B···O24S[g]	2.980(3)	143

[a] -2+x,-2+y,z; [b] -1+x,-1+y,z; [c] 1+x,1+y,z; [d] x,1+y,z; [e] -x,-1+y,z; [f] 2+x,1+y,z; [g] x, y, z; [h] x,y,-1+z; [i] 1+x,y,z; [j] x,-1+y,1+z; [k] -1+x,y,z.

Table S6. Selected hydrogen bonds for compounds **3·6H$_2$O** and **4·3H$_2$O**.

Compound	D–H···A	*d*(D–H···A) / Å	∠(D–H···A) / °
**3·6H$_2$O**	O13–H13A···O32	2.867(4)	168(4)
	O23–H23B···O42	2.886(4)	173(6)
	O2D1–H2D1···O22S[a]	2.717(6)	152
	N1–H1B···O14S[b]	3.035(4)	173
	N2–H2A···O22S[a]	3.035(4)	173
	N2–H2A···O25S[a]	2.896(15)	165
	N3–H3A···O12S[b]	2.985(5)	144
	N4–H4B···O24S[c]	3.138(6)	148
	N4–H4B···O26S[c]	3.218(17)	173
**4·3H$_2$O**	O1D–H1D···O14S[d]	2.666(17)	120
	O2D–H2D···O23S[b]	2.730(16)	150
	N1–H1A···O11S[d]	2.849(16)	168
	N2–H2B···O24S[b]	2.894(15)	165
	N3–H3B···O12S[b]	2.918(15)	172
	N3–H3B···O2S[b]	3.048(11)	114
	N4–H4A···O21S[e]	3.067(12)	120
	N4–H4A···O21S[e]	2.883(14)	166

[a] -1+x,y,-1+z; [b] x, y, z; [c] -1+x,-1+y,-1+z; [d] 1-x,-1/2+y,-z; [e] 1-x,-1/2+y,1-z.

Table S7. Selected hydrogen bonds for compounds **5·4H$_2$O** and **6·5CH$_3$OH.**

**5·4H$_2$O**	O1D–H1D···O11S[a]	2.671(7)	173
	O2D–H2D···O12S[b]	2.798(9)	169(16)
	N1–H1A···O12S[c]	2.995(8)	157(12)
	N2–H2A···O13S[c]	2.881(8)	144(12)
	N2–H2B···O11S[b]	3.019(8)	171(14)
**6·5CH$_3$OH**	O2D–H2D···O12[d]	2.776(4)	169
	N2–H2B···O1D[e]	2.902(4)	173
	O1D–H1D···O3S[c]	3.109(4)	138
	O1D–H1D···O4S[c]	2.736(3)	156
	N1–H1B···O2S[b]	3.045(4)	146
	N1–H1C···O4S[c]	3.046(4)	169
	N2–H2A···O3S[d]	2.900(4)	126

[a] 1+x,y,z; [b] x,y,z; [c] 1-x,y,z; [d] -x,1/2+y,1/2-z; [e] -1-x,1/2+y,1/2-z.

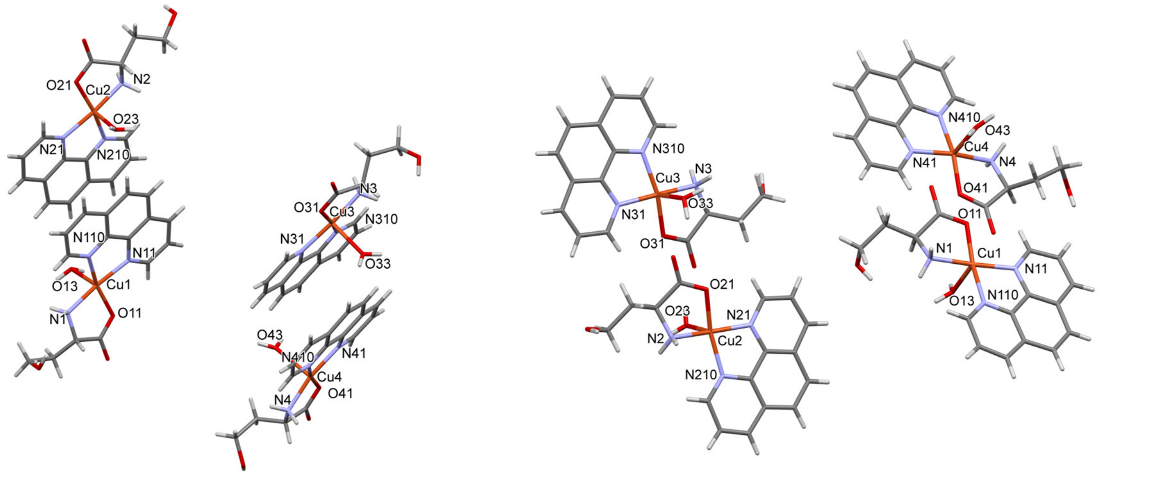

1·5H₂O **2·12H₂O**

Figure S2. The asymmetric unit of **1·5H₂O** and **2·12H₂O**. Crystallization water molecules and sulfate are omitted for clarity.

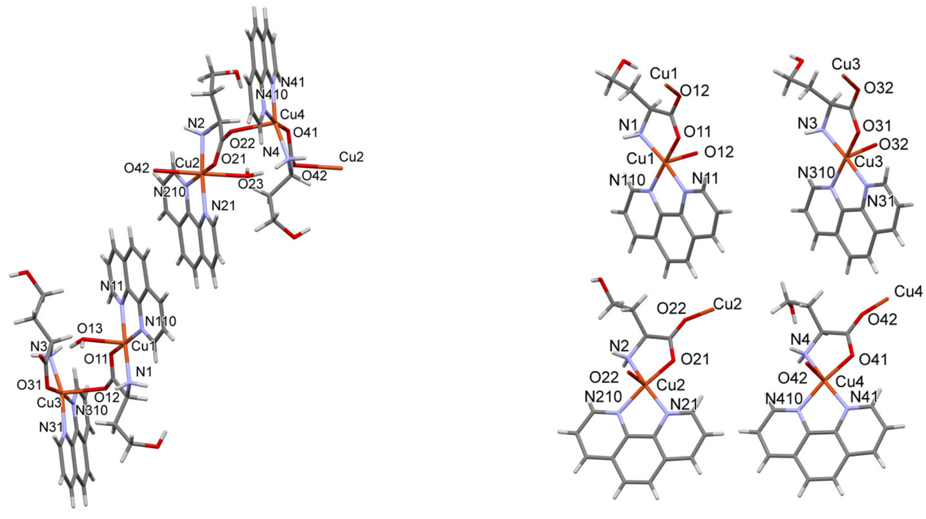

3·6H₂O **4·3H₂O**

Figure S3. The asymmetric unit of **3·6H₂O** and **4·3H₂O**. Crystallization water molecules and sulfate are omitted for clarity.

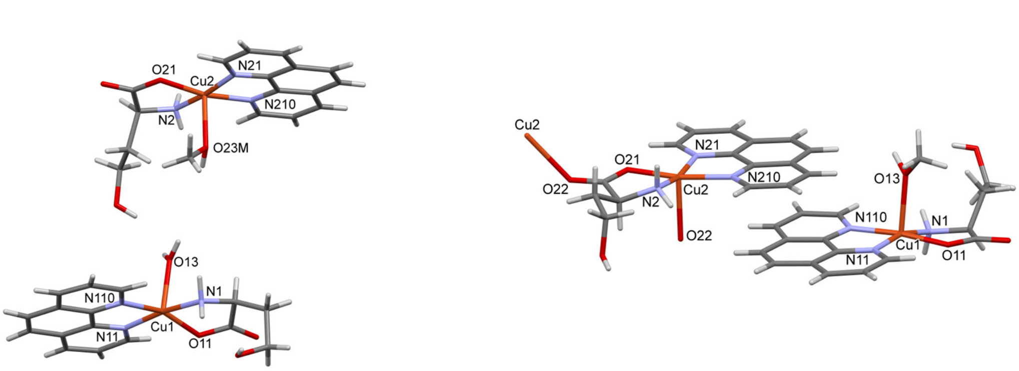

5·4H₂O **6·5CH₃OH**

Figure S4. The asymmetric unit of **5·4H₂O** and **6·5CH₃OH**. Crystallization water/methanol molecules and sulfate are omitted for clarity.

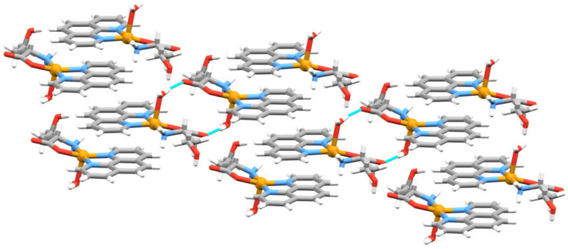

1·5H_2O

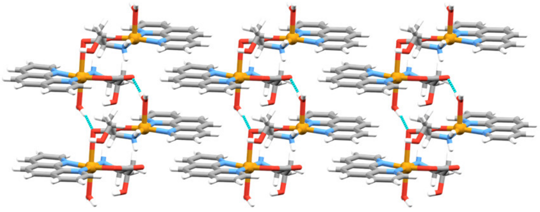

2·12H_2O

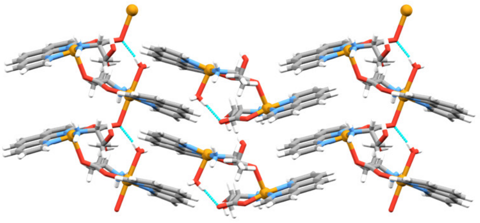

3·6H_2O

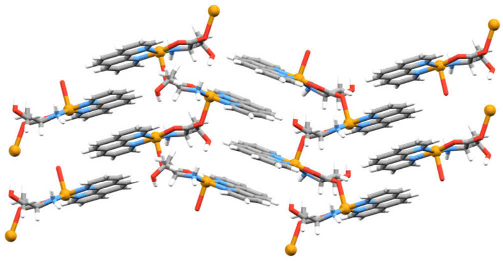

4·3H_2O

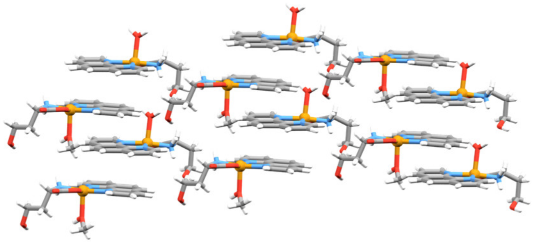

5·4H_2O

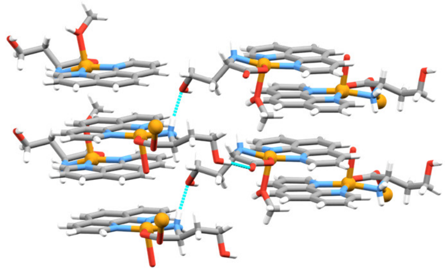

6·5CH_3OH

Figure S5. Hydrogen bonds and coordinative bridging between π-stacked 1D pillars.

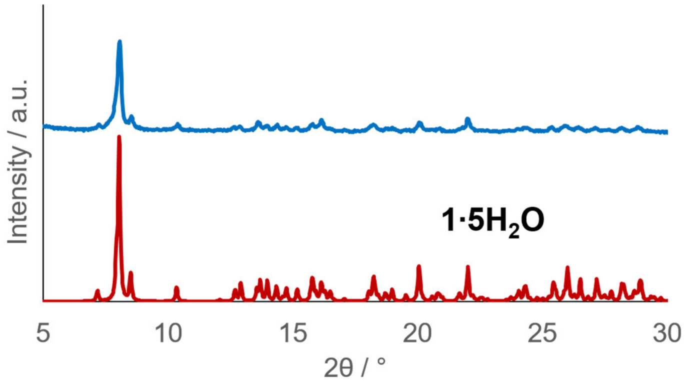

Figure S6. Experimental (blue) PXRD pattern obtained from synthesis and PXRD patternt calculated from crystal structure of **1·5H$_2$O** (red).

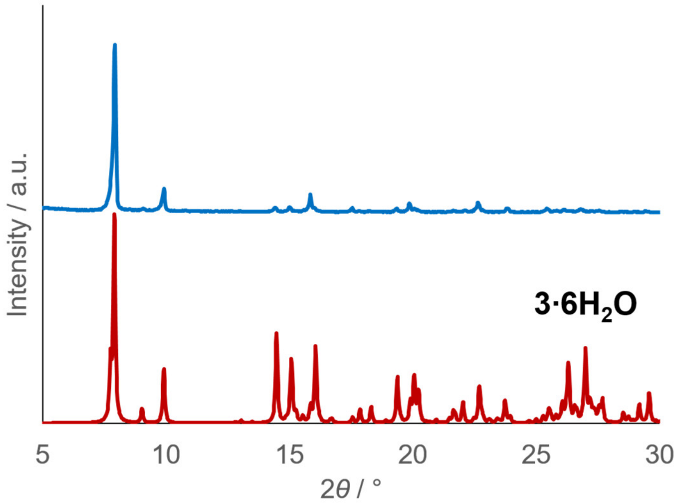

Figure S7. Experimental (blue) PXRD pattern obtained from synthesis and PXRD patternt calculated from crystal structure of **3·6H$_2$O** (red).

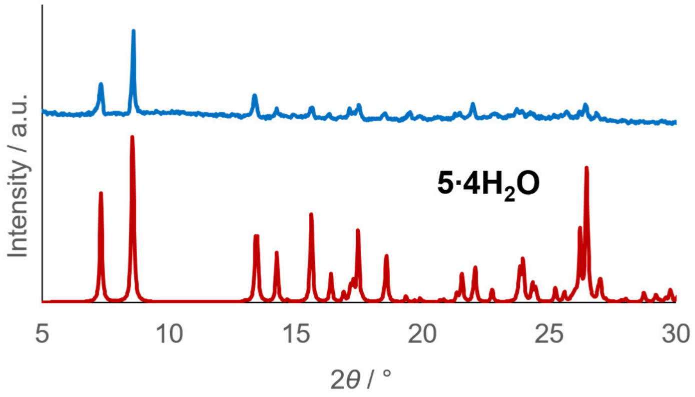

Figure S8. Experimental (blue) PXRD pattern obtained from synthesis and PXRD patternt calculated from crystal structure of **5·4H$_2$O** (red).

Proliferation assays

The experiments were performed on three human cell lines, including HCT 116 (colon carcinoma), H 460 (lung carcinoma), MCF-7 (breast carcinoma), in line with previously published experimental procedures (refs. [26] and [27] of the paper). Cells were grown in DMEM medium with the addition of 10% fetal bovine serum (FBS), 2 mM L-glutamine, 100 U/mL penicillin and 100 µg/mL streptomycin and cultured as monolayers at 37 °C in a humidified atmosphere with 5% CO_2. The cells were seeded on standard 96-well microtiter plates and allowed to attach for 24 hours. The next day, the test compounds were added in five serial 10-fold dilutions. Cell viability was assessed after 72 hours of incubation using the MTT assay, a colorimetric assay system that detects dehydrogenase activity in viable cells. The absorbance, measured on a microplate reader at 570 nm, is directly proportional to cell viability. The percentage of growth (PG) of the cell lines was calculated. The results obtained are expressed as an IC_{50} value, which represents the concentration of the compound required for 50% growth inhibition. The IC_{50} values were calculated from the concentration-response curve using linear regression analysis by adjusting the test concentrations giving PG values above and below the reference value (i.e. 50%). If all tested concentrations result in PG values above the respective reference value, the highest tested concentration is marked with a ">" sign as the default value. Each test was performed in quadruplicate in at least two individual tests.

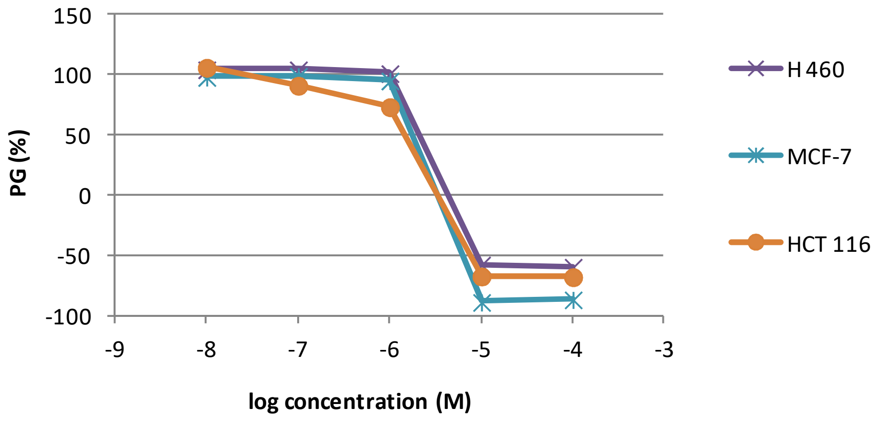

Figure S9. Concentration-response profiles for **3·6H2O** tested *in vitro* on HCT116, MCF-7 and H 460 cell lines.
